# Sexual and reproductive health services access and provision in Cambodia during the COVID-19 pandemic: a mixed-method study of urban–rural differences

**DOI:** 10.1186/s12978-023-01614-y

**Published:** 2023-05-15

**Authors:** Mengieng Ung, Sze Tung Lam, Sovannary Tuot, Pheak Chhoun, Virak Prum, Michiko Nagashima-Hayashi, Pearlyn Neo, Manar Marzouk, Anna Durrance-Bagale, Davide De Beni, Siyan Yi, Natasha Howard

**Affiliations:** 1 Saw Swee Hock School of Public Health, National University of Singapore and National University Health System, 12 Science Drive 2, Singapore, 117549 Singapore; 2 Department of Global Health and Development, London School of Hygiene and Tropical Medicine, 15-17 Tavistock Place, London, WC1H 9SH UK; 3 KHANA Center for Population Health Research, #33, Street 71, Phnom Penh, Cambodia; 4 Center for Global Health Research, Touro University California, Vallejo, CA USA; 5 United Nations Population Fund, Asia and Pacific Regional Office, 4th Floor, UN Service Building, Bangkok, 10200 Thailand

**Keywords:** Sexual and reproductive health, COVID-19, Access, Women, Adolescents

## Abstract

**Background:**

The COVID-19 pandemic pushed governments worldwide to implement unprecedented mitigation measures, including safe-distancing, lockdowns, disruption of non-essential services, border closures and travel restrictions, with both potential to affect rural and urban service-users differently and unintended consequences including reductions in sexual and reproductive health (SRH) services. We aimed to explore rural–urban differences in progress and challenges in SRH services provision in Cambodia, particularly during initial months of the COVID-19 pandemic.

**Methods:**

We used a mixed-methods study design, including a household survey of 423 adolescents and women aged 18–49 and semi-structured interviews with 21 healthcare providers. We analysed survey data using multivariable logistic regression to identify associations between rural–urban setting and contraceptive perceptions or access. We analysed interview data thematically.

**Results:**

Rural–urban residence was significantly associated with reported perceptions about and access to contraceptives. Rural participants had higher odds of stating it was possible to change contraceptive methods early in the COVID-19 pandemic, compared with urban participants. Qualitative data showed that although SRH services continued, health-workers faced differential challenges in rural and urban areas, e.g. service-users not attending due to job losses in urban areas and not complying with safe-distancing and mask-wearing requests in rural areas.

**Conclusions:**

COVID-19 and inadequate mitigation responses differentially affected rural and urban SRH service providers and service-users, exacerbating existing socioeconomic stressors while adding new fears of infection, transport constraints, and reduced livelihoods. Added financial support could help mitigate challenges in both rural and urban areas.

## Introduction

Since the COVID-19 pandemic began, countries have adopted mitigation measures, including safe distancing, reducing non-essential services, lockdowns, border closures, and travel restrictions to curb disease spread. Unintended consequences of these measures have included economic disruption, job and education losses, and overwhelmed health systems due to people seeking pandemic-related services [2, 14, 32]. Redirecting healthcare services toward pandemic responses caused service gaps elsewhere, including sexual and reproductive health (SRH) services provision [11, 33]. These gaps were exacerbated by movement restrictions and social isolation, resulting in decreased health services use. Access to family planning can be reduced or disrupted during crises, particularly in resource-limited countries, as can the number of pregnant women seeking antenatal care (ANC), postnatal care (PNC), and facility-based deliveries [20]. This gap in essential care can put women and adolescent girls at greater risk of adverse outcomes such as stillbirth, spontaneous abortion, unwanted pregnancy, or even maternal death [19]. Insufficient access to SRH care and contraceptives was found to increase sexually-transmitted infections, including human immunodeficiency virus (HIV) in Guinea, Liberia, and Sierra Leone during the 2014–2015 Ebola epidemic [16].

Cambodia’s historically poor SRH indicators, including a 2000 maternal mortality ratio of 488 per 100,000 live births that was among the highest in the Southeast Asia region, improved significantly with government-led interventions [10]. This was reflected in a reduced maternal mortality ratio of 168/100,000 by 2016 [31]. However, progress is fragile and barriers to accessing SRH services, particularly contraceptives, could reverse Cambodia's progress [10]. Thus, the onset of the COVID-19 pandemic raised concerns nationally.

Despite a growing body of research focusing on SRH, few studies target young people aged 18–24, resulting in significant knowledge gaps. Adolescent SRH is of particular concern in Cambodia due to rapid increases in the adolescent population [9],Cambodia has the largest proportion of adolescent and young adult population within Southeast Asia [21]. Young people and women are deemed vulnerable to poor SRH in the region [28]. They face risks of abuse, may engage in unprotected sexual intercourse, become pregnant before their bodies are sufficiently developed or with insufficient spacing between pregnancies, or be unable to seek family or professional support due to stigma. Furthermore, with limited SRH coverage in school curricula, many adolescents lack adequate information for informed decision-making, potentially leading to adverse SRH outcomes [29]. Thus, SRH-related knowledge and practices among Cambodian young people and women should be assessed, particularly during the COVID-19 pandemic, to identify potential service challenges and gaps.

We aimed to explore progress and challenges in SRH services provision in Cambodia, especially during the first six months of the COVID-19 pandemic and identify any rural–urban differences. Objectives were to: (i) assess reported perceptions and experiences of SRH needs and service use among young people of both genders (aged 18–24) and women (aged 25–49) in urban and rural areas of Cambodia; (ii) explore SRH provider perspectives of SRH service effectiveness and use; and (iii) inform government and partner agency support for SRH services during the pandemic.

## Methods

### Study design

We used a concurrent mixed-method design, including a cross-sectional household survey and semi-structured key informant interviews (KIIs) with SRH service providers in one urban (i.e. Phnom Penh city) and two rural provinces (i.e. Kampong Cham and Mondulkiri).

### Sampling and recruitment

Survey. We used 2020 population projection data from the National Institute of Statistics [13] to calculate survey sample size, assuming a 95% confidence interval (95% CI) and 10% non-response rate, giving a minimum total required sample size of 425 participants. We employed multi-stage random sampling, selecting participating households from urban (i.e. Phnom Penh) or rural (i.e. Kampong Cham, Mondulkiri) settings and excluding provincial towns in each rural province from the sampling frame (provincial towns are urban areas, administratively equivalent to district level, where provincial departments are located). Household eligibility was based on including a male or female youth (aged 18–24 years) or women aged 25–49 years. Although the United Nations defines youth as persons aged 15–24 years, we did not include those under 18 years due to ethical concerns, practicalities of getting informed consent from guardians, and previous research experience in Cambodia showing adolescents under 18 years yielded little SRH information. Participant eligibility included: (i) being married, as this was a legal consideration when asking about SRH in Cambodia,(ii) residing in the community for the last six months; (iii) being psychologically able to participate in a survey without difficulties; (iv) able and willing to provide written informed consent; and (v) able to communicate verbally in Khmer, Cambodia’s official language. First, we randomly selected operational districts, health centres, and villages within selected health centre catchments. Second, with support from village chiefs and village health support groups, we randomly selected 423 participants, obtaining a response rate of 100% (Table 1). Participants each received US$5 for their time.

**Table 1:** Sociodemographic characteristics stratified by residence (n = 423)

Variable	Urban, n (%)	Rural, n (%)	Total, n (%)
Age and sex			
Young men	48 (23)	31 (15)	79 (19)
Young women	60 (28)	79 (37)	139 (33)
Older women	103 (49)	102 (48)	205 (48)
Ethnicity			
Khmer	209 (99)	134 (63)	343 (81)
Vietnamese	2 (1)	0 (0)	2 (1)
Phnong	0 (0)	78 (37)	78 (18)
Participant education			
No education	12 (6)	28 (13)	40 (9)
Primary (grades 1–6)	52 (25)	73 (34)	125 (30)
Secondary (grades 7–9)	71 (34)	52 (25)	123 (29)
High school (grades 10–12)	43 (20)	48 (23)	91 (22)
Bachelor’s or more	33 (15)	11 (5)	44 (10)
Marital status			
Single (never married)	85 (40)	64 (30)	149 (35)
Co-habiting unmarried	1 (0)	0 (0)	1 (0)
Married	121 (58)	139 (66)	260 (62)
Divorced/separated/widowed	4 (2)	9 (4)	13 (3)

Interviews. We conducted 21 KIIs with providers at the same time, three at provincial health department level, two at operational district level, three at referral hospital level, seven at health centre level, and six at village level (Table 2). KII eligibility criteria included: (i) providing SRH services for at least six months prior to interview; (ii) able and willing to provide written informed consent; and (iii) able to communicate in Khmer. Participants each received US$10 for their time.

**Table 2:** Contraceptive access and use by urban–rural setting, gender, and age group (n = 423)

Variable	Urban				Rural			
	Young men	Young women	Older women	Total	Young men	Young women	Older women	Total
	n (%)	n (%)	n (%)	n (%)	n (%)	n (%)	n (%)	n (%)
Current contraceptive method								
No method	44 (92)	42 (70)	37 (36)	123 (58)	29 (94)	46 (58)	40 (39)	115 (54)
Copper IUD	48 (100)	1 (2)	2 (2)	3 (1)	0 (0)	0 (0)	1 (1)	1 (1)
Hormonal IUD	0 (0)	1 (2)	1 (1)	2 (1)	0 (0)	1 (1)	0 (0)	1 (1)
Implant	0 (0)	0 (0)	1 (1)	1 (1)	0 (0)	0 (0)	1 (1)	1 (1)
Injectable	0 (0)	1 (2)	3 (3)	4 (2)	0 (0)	6 (8)	11 (11)	17 (8)
Male/female condom	3 (6)	1 (2)	5 (5)	9 (4)	0 (0)	1 (1)	2 (2)	3 (1)
Pills	0 (0)	7 (12)	18 (18)	25 (12)	1 (3)	17 (22)	24 (24)	42 (20)
Sterilisation	0 (0)	0 (0)	5 (4.5)	5 (2)	0 (0)	0 (0)	5 (5)	5 (2)
Withdrawal/natural methods	1 (2)	8 (13)	51 (50)	61 (29)	1 (3)	14 (18)	24 (24)	39 (19)
Access to contraceptives comparing 2020 to 2019 (N = 185)								
Much easier	0 (0)	2 (11)	0 (0)	2 (2)	0 (0)	1 (3)	5 (8)	6 (6)
Somewhat easier	0 (0)	0 (0)	9 (14)	9 (10)	0 (0)	5 (15)	7 (11)	12 (12)
No difference	4 (100)	6 (33)	31 (47)	41 (47)	0 (0)	14 (42)	10 (16)	24 (25)
Somewhat more difficult	0 (0)	1 (6)	0 (0)	1 (1)	N/A	N/A	N/A	N/A
Much more difficult	0 (0)	9 (50)	26 (39)	35 (40)	2 (100)	13 (39)	40 (65)	55 (57)

### Consent process

We provided a study information sheet to each potential participant, summarising the protocol, potential risks and benefits of participation, and how confidentiality and sensitivity were handled. After addressing any queries, data collectors obtained written informed consent prior to survey or key informant interview.

### Data collection

Survey. We developed a structured survey questionnaire based on previously validated KHANA survey tools, modifying questions based on information requirements and study populations (i.e., young and older women, young men). The questionnaire included three sections: (i) socioeconomic and demographic characteristics, (ii) general health (i.e. perceived physical health, quality of life, depression, and anxiety) and (iii) SRH and maternal health (i.e. access to contraceptives, pregnancy, delivery, access to ANC and PNC). Participants answered questions on the pre-pandemic (December 2019) and early-pandemic periods (July–August 2020). The questionnaire was developed in English, translated into Khmer, and back-translated into English by another translator to ensure original 'content and spirit' were maintained. Once finalised, the questionnaire was transferred to tablets running KoBoToolbox software. Each questionnaire took approximately 45 min to complete. Experienced enumerators collected data July–August 2020 after piloting the questionnaire face-to-face with seven women aged 18–49 years recruited from Phnom Penh to ensure wording and contents were socio-culturally suitable and comprehensible (e.g. content, format, length, language appropriateness).

Interviews. We developed the interview guide in English, and translated it into Khmer, in consultation with national and regional experts to capture key challenges to SRH services provision during the pandemic. Experienced interviewers, trained in qualitative methods, conducted face-to-face interviews with health providers at their health facilities. Interviews were conducted in July–August 2020, concurrently with survey data collection. Each interview took approximately 45–60 min, was audio-recorded, transcribed in Khmer, and translated into English by the research team. Personal information and identifiers were removed from transcripts and field notes to ensure anonymity and confidentiality.

### Analysis 

Survey. We analysed survey data using Stata 15 (StataCorp, Texas, USA). We used descriptive statistical tests to compute means and standard deviations for numerical variables and frequencies for nominal and ordinal variables. We used χ^2^ test or Fisher's exact test (i.e. when cell counts were smaller than five) for categorical variables and Student's t-test for continuous variables. Two-sided p-values < 0.05 were deemed statistically significant. Significant χ^2^ test results with a degree of freedom ≥ 2, or category with adjusted residual ≥ 1.96, were treated as contributing to statistical significance. We used multivariable logistic regression to analyse associations between age, setting, household income, contraceptive perceptions, and changes in access to health services and contraceptives. Predictor variables were age group (18–24 or 25–49), setting (urban or rural), and household income (i.e. lower < US$200, middle US$200–614, higher > US$615). Logistic regression analyses of binary outcome variables included: (i) perceived safety of contraception use during the pandemic; (ii) possibility of changing contraceptive method during the pandemic; (iii) necessity of contraceptives in preventing unwanted pregnancy during the pandemic; and (iv) access to contraceptives.

Interviews. As survey and interview data were collected and analysed concurrently, qualitative interview findings primarily served to add nuance to quantitative survey findings. We analysed qualitative data thematically using deductive and inductive coding as described by Braun and Clarke [1]. This consisted of data familiarisation, generating initial codes, searching for themes, reviewing, defining, and agreeing theme names, and writing up the analytic narrative and extracted quotes. We based deductive codes on interview guide topics, while also identifying inductive codes and themes from the data. Data reporting adhered to COREQ standards [26].

### Ethics 

We obtained ethics approval from the National Ethics Committee for Health Research (NECHR-162) in Cambodia and the National University of Singapore Saw Swee Hock School of Public Health departmental ethics committee (SSHSPH 093-1).

## Findings

### Quantitative survey findings

#### Descriptive analysis

Table 1 shows sociodemographic characteristics of 423 participants, including 139 young women, 79 young men, and 205 older women aged 25–49. Most participants (81%) were Khmer, 1% were Vietnamese, and 18% were Phnong—all living rurally. Most had completed primary (30%), secondary (29%), high school (22%), or university (10%), while 9% had no formal education (6% urban versus 13% rural). Educational attainment varied by location, e.g. more university degrees among urban participants (15% urban versus 5% rural), as well as gender and age, with 43% of young men having finished high school, 32% of young women having finished secondary education, and 43% of older women having finished primary education. Over 60% were married, although over 91% of young men were single.

Table 2 shows urban–rural differences in contraceptive methods used. Main differences included the proportion of participants who used no contraception (i.e. urban 58%, rural 54%), used injectables (i.e. urban 2%, rural 8%), condoms (i.e. urban 4%, rural 1%), pills (i.e. urban 12%, rural 20%), and natural methods (i.e. for rhythm, 11% urban versus 7% rural; for withdrawal 18% urban versus 12% rural). In considering access during COVID-19 pandemic compared with previously, responses were mixed with higher percentages of rural participants describing both increased ease (18% versus 12%, respectively) and increased difficulty accessing contraception (i.e. 57% versus 40%, respectively).

#### Multivariable analysis

Table 3 shows that rural households had over twice the adjusted odds (AOR 2.49; 95% CI 1.32–4.91) of reporting that changing contraceptive methods during the pandemic was possible compared to urban participants. However, rural participants also had approximately three times higher adjusted odds of both reporting contraceptive access as more difficult (AOR 2.92; 95% CI 1.39–6.13) and easier (OR3.29; 95%CI 1.21–8.92) in 2020 than in 2019 as compared with urban participants.

**Table 3:** Associations between rural setting and perceptions and reported behaviours during the COVID-19 pandemic compared to pre-pandemic (n = 423)

Predictor variables	Adjusted odds ratio of outcome (95% CI)				
	Contraception is safe (N = 326)	It is possible to change contraception (N = 307)	Contraception is necessary to prevent unwanted pregnancy (N = 378)	Access to contraceptives (N = 185)	
				Harder	Easier
Rural (ref. urban)	0.83 (0.43, 1.62)	2.49 (1.32,4.91)**	1.17 (0.44, 3.29)	2.92 (1.39, 6.13)**	3.29 (1.21, 8.92)*

	Physical aggression	Increased tension in family	Increased depression	Increased tension toward children	
	1.89 (0.84, 4.58)	1.20 (0.75, 1.92)	1.39 (0.88, 2.19)	0.98 (0.46, 2.06)	

*CI* confidence interval

*p < 0.05; **p < 0.01

Rural participants had no significant differences in adjusted odds of reporting increased physical aggression toward household members (AOR 1.89; 95% CI 0.84–4.58), family tension (AOR 1.20; 95%CI 0.75–1.92), depressive symptoms (AOR 1.39; 95% CI 0.88–2.19), or tension towards children (AOR 0.98; 95% CI 0.46–2.06) as compared to urban participants.

### Qualitative interview findings

#### Participant characteristics 

Table 4 summarises characteristics of our 21 key informants, 9 in urban Phnom Penh and 12 in rural provinces (i.e. 7 in Kampong Cham and 5 in Mondulkiri). In total, 15 were health-workers (e.g. at provincial health department, operational districts, referral hospitals, health centres) and 6 were community health support group members. More than 80% of participants were women (i.e. 89% in urban and 75% in rural areas). Median age was 47 (range 25–64) years and career duration was 10 (range 3–25) years. No urban–rural differences were noted in gender, age, or years of service.

**Table 4:** Qualitative interviewee characteristics (n = 21)

ID	Province	Facility level	Role	Gender
3R	Kampong Cham	Provincial Health Department	Maternal and child healthcare senior manager	Male
5R	Kampong Cham	Operational District	Maternal and child healthcare senior manager	Male
7R	Kampong Cham	Referral Hospital	Obstetrics senior manager	Female
12R	Kampong Cham	Health Centre	Sexually transmitted infections consultant	Female
16R	Kampong Cham	Community	Community health support group	Male
20R	Kampong Cham	Community	Community health support group	Female
13R	Kampong Cham	Health Centre	Obstetrics senior manager	Female
1R	Mondulkiri	Provincial Health Department	Maternal and child healthcare senior manager	Female
6R	Mondulkiri	Referral Hospital	Obstetrics senior manager	Female
14R	Mondulkiri	Health Centre	Obstetrics senior manager	Female
15R	Mondulkiri	Health Centre	Midwife	Female
17R	Mondulkiri	Community	Community health support group	Female
2U	Phnom Penh^a^	Provincial Health Department	Maternal and child healthcare senior manager	Female
4U	Phnom Penh^a^	Operational District	Maternal and child healthcare senior manager	Male
8U	Phnom Penh^a^	Referral Hospital	Midwife	Female
9U	Phnom Penh^a^	Health Centre	Midwifery senior manager	Female
10U	Phnom Penh^a^	Health Centre	Obstetrics	Female
11U	Phnom Penh^a^	Health Centre	Midwife	Female
18U	Phnom Penh^a^	Community	Community health support group	Female
19U	Phnom Penh^a^	Community	Community health support group	Female
21U	Phnom Penh^a^	Community	Community health support group	Female

^a^Urban setting

#### Thematic findings

We included one deductive theme (i.e. SRH services access and use) and two inductive themes (i.e. staffing and salary reductions; health and risk communication) that best described our data. While qualitative interviews were primarily intended to clarify and triangulate survey results, we noted that most interviewees focused primarily on their own experiences as SRH service providers during the initial months of COVID-19 and have reported these findings accordingly.

#### SRH services access and use

Views on the impact of the pandemic on access and use of SRH services varied. Participants indicated that rural areas were more affected by mitigation measures than urban areas, as existing transport shortages to connect households to nearby health centres were exacerbated by the public transport ban. However, some interviewees indicated that services for women and adolescents remained relatively unaffected. A rural interviewee from Kampong Cham province indicated that health providers continued to offer maternal and child-related vaccination services and antenatal and postnatal services in village health centres.

"We have all services, prescription services, health check-ups, and maternal and child-related vaccination available. We offer vaccination in the villages and every day at the centre. We offer other services such as delivery, postpartum check-up, birth control methods such as condoms, injections, pills, intrauterine devices, natural contraception like calendar contraception, and condoms." (KII-HC Krouch, Kampong Cham)

Particularly in rural areas, fear of interacting with patients was exacerbated by poor adherence to safe-distancing recommendations among service-users and the concomitant fear of infection. Interviewees described how large numbers of patients and caretakers continued to gather in groups within health facilities.

*"It's really a challenge! Firstly, we are so scared of that public health emergency and afraid we would get that disease from clients because most of them who come here, we don't know if they have that disease or not. Some of them cooperated with us by wearing masks and keep social distancing. Some of them don't wear mask and they are in groups, big groups … when there is someone sick, there are at least three to four persons to take care of a patient. It's hard because of different traditions."* (KII-RH, Mondulkiri)

*"[We are] worried about COVID, afraid it would transmit. Afraid it would transmit in the community. It must be difficult, and we also do not have enough materials. We are the healthcare providers, so we protect ourselves, but the community people come without wearing masks. We tell them to wear masks, but some still do not wear masks. They act normal and are not afraid of the virus"* (KII-HC Krouch, Kampong Cham)

Urban interviewees additionally highlighted how fear of infection hindered service-users from accessing available healthcare services.

*"During this COVID, the numbers of clients have been decreasing, they are afraid to come. The rates of those who are the existing service-users also decrease."* (KH-PHD, Phnom Penh)

Some urban providers reported fewer service-users seeking SRH services from March to May 2020, mainly due to fear of infection and lack of sufficient money. As many service-users lost their employment during the lockdowns, especially those working for the private sector at karaoke clubs and garment factories, they were unable to afford out-of-pocket payments for healthcare services.

*"Most of the women who come here complained about their financial crisis. High unemployment. Many women who work at Karaoke used to come here before, but now they seem not to have any income… we are worried that it would be less and less if the public health emergency keeps going."* (KII-HC Mean Chey, Phnom Penh)

Transportation and physical access to services remained a long-term challenge for SRH in rural areas, even prior to the COVID-19 pandemic. Participants reported that awareness and training sessions were temporarily halted in some villages where unpaved roads prevented transportation of essential supplies and equipment.

*“We cannot do awareness-raising because we are seeking for where the children are to offer vaccination and women who need to get pregnancy check for prenatal and postnatal. Doing that we cannot offer all services because it's a long way, we cannot carry all materials to all communities as they need, the road is an unpaved road, it's difficult to trave*l." (KII-HC Keo Seyma, Mondulkiri)

#### Staffing and salary reductions

Health-worker experiences during the initial phase of the pandemic varied. Some rural providers reported being overwhelmed by the number of service-users and insufficient medical staff, while others reported a decrease in their workload. Reduced staffing and services made it difficult for service-users to access required SRH services, and if services were available, service-users could expect delays or interruptions due to staffing shortages.

*"Lack of staff…because of the epidemic, we sent two staff to the border. So when there are many patients, we face difficulties, we are so busy… We provide service to everyone, but it is a bit slow."* (KII-HC Dak Dam, Mondulkiri)

All providers described feeling overwhelmed by the lack of social and professional support. They reported being quarantined away from their families, which negatively affected their psychological wellbeing, consequently predisposing some to depression and anxiety. On a professional level, the COVID-19 pandemic decreased opportunities for training and supervision, leading to a decrease in professional support and training that was particularly noted by rural participants.

*'We are always affected by COVID. It's also been decreasing related training because we are not allowed to gather as big group, and the supervision activities also reduced, and the patients have been decreasing too."* (KII-PHD, Kampong Cham)

Some rural health-workers also mentioned loss of income due to the cessation of secondary activities such as evaluations and training workshops. Supplementary income from activities organised by development partners provided a significant portion of health-workers’ monthly wages, so this was a significant loss at a time when salary disbursements were also affected by COVID-19 related staffing shortages.

*"[I have] no [supplementary] income. It's because the evaluation was stopped, so no income. There is no evaluation program, so the income has been decreased… During the public health emergency, I couldn't attend any workshops, meetings, so no income… [I have lost] about 20% of salary"* (KII-OD Prey Chhor, Kampong Cham)

Most providers reported that working hours had doubled between December 2019 and July 2020, with many indicating their compensation had also increased in accordance with the hours worked. Some attributed the longer working hours to the increased number of service-users. However, others reported seeing fewer patients and therefore being paid less.

*"I got paid less during the March to April (2020) period."* (KII-HC Prek Pou, Kampong Cham)

While this pattern was not consistent, it appeared that the pandemic contributed to increased service use in some rural facilities and reductions in other, primarily urban, facilities.

#### *Health and risk communication*

During the first year of the pandemic, health-workers in rural areas reported the quality of their communication with service-users worsened due to the precautionary measures they took when interacting with service-users, such as wearing facemasks and personal protective equipment. All providers described being more careful around service-users.

*"We are more careful. When clients come, all staff wear masks and wash hands with alcohol, keep hygiene all the time. We wear gloves. Everything we need to be more careful than before…."* (KII-HC Dak Dam, Mondulkiri).

Some rural interviewees reported a lack of collaboration between frontline health-workers and governmental actors, due to preferences for in-person communication and budget constraints.

*"When we need to communicate with village or commune chiefs, we invite them. Sometimes, the village [chief] doesn't update the commune [sub-district] or police, so they contact us. It's difficult because there is no budget to travel to village or commune."* (KII-HC Keo Seyma, Mondulkiri)

Besides their usual clinical duties, providers described the added burden of training and enforcing service-users to wear facemasks and maintain safe-distancing when they attended health facilities.

*"We have always prepared alcohol for hand sanitizer. We tell them to wear masks, and we keep them far from each other one meter, sitting far from each other, and educate them to wear a mask"* (KII-HC Krouch, Kampong Cham).

Both urban and rural interviewees highlighted that public risk communications about COVID-19 did not effectively engage with minoritized communities. While the reasons why were not clarified, several interviewees described perceived discrimination from the government and the public toward minority communities, which risked isolating and depriving these communities of healthcare services during the pandemic. However, some also demonstrated their own lack of awareness in describing these marginalised communities.

*"A lot of Khmer Muslims […] so they said they are not afraid of COVID, they are afraid of not worshipping..."* (KII-OD Mekong, Phnom Penh)

## Discussion

This unique mixed-method examination of sexual and reproductive health services access and use in urban and rural Cambodia in the early months of the COVID-19 pandemic showed urban–rural differences in reported perceptions about and access to contraceptives during the initial phase of the COVID-19 pandemic in Cambodia.

Survey findings showed that, compared to urban counterparts, rural participants had higher odds of suggesting that it was possible to change contraceptive methods and higher odds of reporting that accessing contraceptives was both more difficult and easier during the pandemic. This may be because in July 2020 the COVID-19 situation in Cambodia was well controlled, with relatively few cases (< 400 cases, 0 deaths) compared with neighbouring countries. Most cases were reported in the capital city, while in rural study sites there were no reported COVID-19 cases or movement restrictions. Thus, rural populations still had relatively easy access to contraception, especially condoms and pills that are generally available at local pharmacies [12].

Qualitative findings indicated that SRH services remained roughly at pre-pandemic levels, with rural–urban differences mentioned in access, workload, and remuneration. User-related factors, such as fears of COVID-19 infection in crowded places and reduced overall income, contributed to reduced SRH service accessibility—a finding common in many countries [5]. However, particularly in rural areas those accessing services appeared relatively unconcerned about COVID-19, given providers comments about having to enforce safe-distancing and mask-wearing. Therefore, it is difficult to determine how much fear of contracting COVID-19 versus income loss influenced access in the early period of the pandemic in Cambodia. Additionally, healthcare providers may have facilitated long-term dispensing of SRH pharmaceuticals and products to reduce physical presence in pharmacies and health facilities, as noted elsewhere [34].

The pandemic put health-workers globally under unprecedented strain, demanding they balance personal and family physical and mental healthcare needs with those of patients, while working under extreme pressure with constrained resources [6]. Many qualitative interviewees noted reductions in salaries during lockdown, along with workload disruptions. Lockdown was implemented from 14 April to 5 May 2021 in Phnom Penh [27] and subsequently extended to provinces. Unpredictable working hours, task shifting, and reductions in income have been shown to worsen mental health and exacerbate healthcare staffing shortages [4, 7, 15]. The COVID-19 pandemic also presented challenges to healthcare education [17], with many interviewees complaining about reduced training opportunities, which could affect workforce quality [22, 30]. However, while our interviewees indicated some concerns—particularly related to psychological wellbeing and salary—rural–urban differences were minimal and services did not appear overly affected at this early stage of the pandemic.

The Cambodian health system generally recognised that pandemic-related constraints, such as quarantines and travel restrictions, could challenge community structures and access to quality SRH services. An important aspect in any governmental response to crises is communication [25]. Pandemic risk communication must incorporate community engagement, especially given evolving technical knowledge of COVID-19 and its epidemiology, Cambodia’s socio-cultural diversity, and a dynamic global media landscape [8]. Survey participants did not mention health or other communication initiatives, while qualitative interviewees mentioned both its importance and gaps. Effective engagement with marginalised and disenfranchised communities must be considered when developing an effective risk communication strategy for Cambodia. To address these vulnerabilities in this or future pandemics, health actors should consider potential urban–rural differences in access and provision and provide risk communication and engagement that includes marginalised communities, funding and technical support to public health facilities, as most participants reported seeking services in the public sector,and psychosocial support for frontline health-workers who reported facing uncertainty and overwhelm as the pandemic progressed.

### Limitations

Several limitations should be noted. First, we focused on women and young people, so older men's views are not directly represented. Second, we treated women as homogenous in the survey due to resource and access constraints, despite studies suggesting that women and adolescents with additional potential vulnerabilities, such as those belonging to indigenous populations, living with HIV, or with pre-existing physical or mental disabilities, would be disproportionately disadvantaged by pandemic-related disruptions to SRH services. Third, cross-sectional surveys present prevalence and cannot identify causality. Fourth, this survey included approximately 400 households, so findings should not be generalised. However, inclusion of qualitative interviews allowed additional exploration of issues that help inform our findings. Fifth, we were restricted to recruiting interview participants who could communicate in Khmer or English, due to the language capacities of interviewers, so minoritized people who did not speak Khmer could not be included. Finally, the situation is dynamic and has continued to change since data were collected.

## Conclusions

COVID-19 and initial mitigation responses negatively affected SRH service access and provision in Cambodia, with rural–urban differences predominantly exacerbating existing socioeconomic stressors while adding new fears of infection risk, such as transport constraints between rural villages, lack of health-worker funding and training, and insufficient safe-distancing and mask-wearing among service-users—particularly in rural areas.

## Data Availability

Anonymised data supporting the findings of this study are available from the corresponding author, upon reasonable request.
